# Dietary restraint, binge eating and adherence to an adolescent intensive behavioural weight management intervention: a Fast Track to Health sub-study

**DOI:** 10.1007/s40519-026-01876-y

**Published:** 2026-06-04

**Authors:** Eve T. House, Natalie B. Lister, Louise A. Baur, Sarah P. Garnett, Megan L. Gow, Cathy Kwok, Hiba Jebeile

**Affiliations:** 1 Sydney Medical School, The University of Sydney, Westmead, NSW Australia; 2 Institute of Endocrinology and Diabetes, The Children’s Hospital at Westmead, Westmead, NSW Australia; 3 Weight Management Services, The Children’s Hospital at Westmead, Westmead, NSW Australia; 4 Kids Research, The Children’s Hospital at Westmead, Westmead, NSW Australia; 5 The George Institute for Global Health, UNSW Sydney, Sydney, NSW Australia

**Keywords:** Dietary restraint, Dieting, Adolescent, Obesity, Dietary intervention

## Abstract

**Purpose:**

Dietary restraint often increases or remains unchanged following adolescent weight management interventions. It is unknown whether dietary restraint change in such interventions is associated with binge eating risk or intervention adherence. This study examined associations between (1) dietary restraint, measured using the Eating Disorder Examination Questionnaire (EDE-Q) and Dutch Eating Behaviour Questionnaire (DEBQ); (2) changes in dietary restraint and binge eating; and (3) changes in dietary restraint, anthropometry, and adherence throughout a weight management intervention.

**Methods:**

Secondary data analysis of a clinical trial of behavioural weight management interventions involving adolescents (13–17 years) with obesity and related complications. Associations between dietary restraint, binge eating, and BMI z-score were examined using Pearson or Spearman’s rank correlation. Differences in dietary restraint change between adherers and non-adherers were examined by independent samples t-tests or Mann–Whitney U tests.

**Results:**

141 adolescents (14.8 years, 49.6% female) were recruited; 136 had baseline, 130 week-4, 120 week-16, and 92 week-52 data included in analyses. EDE-Q and DEBQ restraint scores were correlated at all timepoints (*r* = 0.442–0.579, all *p* < 0.001). Changes in dietary restraint and binge eating from baseline to week-4 and week-16 were not correlated. Week-52 change in DEBQ dietary restraint was associated with change in binge eating (*r* = 0.347, *p* < 0.001). Correlations between changes in dietary restraint and BMI z-score and differences in change in dietary restraint between adherers and non-adherers were not significant.

**Conclusion:**

The findings do not provide robust evidence of dietary restraint as a marker of adherence or binge eating risk during a behavioural weight management intervention.

**Level of evidence:**

Level III: Evidence obtained from well-designed cohort or case–control analytic studies.

**Supplementary Information:**

The online version contains supplementary material available at 10.1007/s40519-026-01876-y.

## Introduction

‘Dietary restraint’ refers to the cognitive effort directed towards attempts to limit dietary intake, regardless of actual intake, and has been implicated in models of eating disorder (ED) development, particularly bulimia nervosa and binge eating disorder [1, 2]. The context in which dietary restraint occurs plays a crucial role in its impact. When practised as part of healthy self-regulation, dietary restraint may support individuals to manage their food intake effectively. However, when driven by unhealthy self-regulation, it may reflect rigid, all-or-nothing thinking and contribute to disordered eating behaviours [1].

Evidence for dietary restraint as an ED risk factor has primarily come from longitudinal, community-based studies with adolescents and young adults across the weight spectrum [1]. The dual pathway model of ED development posits that societal pressure for thinness and internalisation of this body ideal result in body dissatisfaction. This dissatisfaction may lead to dietary restraint and/or negative affect, both of which are predictive of ED onset, particularly binge eating and/or purging behaviours [2]. Based on this model, we might expect that interventions that result in increases in dietary restraint could lead to increases in these disordered eating outcomes.

Weight management interventions for adolescents with obesity often involve dietary modification, particularly self-regulation of energy intake and/or improvement in diet quality, which may require dietary restraint [1]. It is important to understand how such interventions impact the risk of EDs and disordered eating behaviours. Our 2021 systematic review found that dietary restraint either increased or remained unchanged following paediatric weight management interventions while other disordered eating outcomes improved or remained unchanged [3]. Studies included in our review measured dietary restraint using several different tools, including the Dutch Eating Behaviour Questionnaire (DEBQ) [4] and Eating Disorder Examination Questionnaire (EDE-Q) [5]. The DEBQ was designed to characterise eating patterns associated with obesity, while the EDE-Q was designed to identify anorexia nervosa and bulimia nervosa [4, 5]. It is important to understand how different measures of dietary restraint change and their relation to disordered eating outcomes in weight management interventions.

Most interventions included in our 2021 review involved moderate energy restriction, general healthy eating advice and/or a traffic light diet (an approach used in paediatric weight management which classifies foods as ‘red’ [should be limited], ‘yellow’ [consume in moderation], and ‘green’ [everyday choices] [6]). Less is known about the impact of intensive dietary interventions, such as a very low energy diet (VLED) or intermittent energy restriction, on dietary restraint and its interplay with disordered eating outcomes. The Fast Track to Health (Fast Track) randomised controlled trial (RCT) compared two intensive dietary interventions for adolescents with obesity and associated cardiometabolic complications. The Fast Track interventions resulted in a reduction (0.28 estimated marginal mean) in body mass index (BMI) z-score at week-52 with no difference between intervention groups [7]. Both groups had decreased global ED risk scores maintained to week-52, however, there was a transient increase in dietary restraint which returned to baseline levels by week-52 [8, 9]. There is a lack of evidence regarding the impact of intensive dietary interventions on dietary restraint, and the association between changes in dietary restraint and changes in disordered eating behaviours in this context.

This study aimed to examine the association between (1) different measures of dietary restraint used in the Fast Track trial; (2) changes in dietary restraint during the trial and changes in binge eating; and (3) changes in dietary restraint, anthropometric outcomes, and adherence to the intervention.

## Methods

### Study design

Secondary analysis of data from the Fast Track trial was conducted. The trial protocol [10] and primary outcomes [7, 8] have been published. The trial was approved by the Sydney Children’s Hospital Network Human Research Ethics Committee (HREC/17/SCHN/164) and prospectively registered with the Australian New Zealand Clinical Trial Registry (ACTRN12617001630303).

In brief, from 2018 to 2022, adolescents (13–17 years) with obesity and ≥1 associated cardiometabolic complication (e.g. insulin resistance, dyslipidaemia, hypertension) were recruited to participate in Fast Track. Self-report questionnaires—the EDE-Q [5] and Center for Epidemiologic Studies Depression Scale Revised-10-item version (CESDR-10) [11] were used to screen for depression and EDs prior to enrolment. Participants with a current ED diagnosis were ineligible for the trial and referred to appropriate support services. Adolescents took part in a 52-week RCT of intensive behavioural weight management interventions. They underwent a 4-week VLED and then transitioned to their allocated dietary pattern—an intermittent (IER) or continuous (CER) energy-restricted dietary pattern—for the remainder of the trial. The IER dietary pattern involved three days per week of reduced energy intake (600–700 kcal/day) alternated with four days of healthy eating without an energy prescription. The CER dietary pattern involved a consistent daily energy prescription of 1430–1900 kcal/day (dependent on age). Adolescents were transitioned to a weight maintenance plan at study completion, or earlier if they had entered the healthy weight range or reached their personal goal weight. Multidisciplinary support was provided by a study dietitian during a baseline assessment, 12 review appointments, and 9 scheduled supports via phone, text message or email; the study paediatrician at baseline and week-16; with additional support, including from a clinical psychologist, as clinically indicated [7].

### Data collection

#### Demographic information

Participant demographic information and clinical history were collected at baseline.

#### Dietary restraint and binge eating outcomes

Dietary restraint and binge eating outcomes were collected using electronic surveys distributed via REDCap hosted at the University of Sydney, a secure online research data management platform. Questionnaires were completed at baseline, week-4, week-16 and week-52.

Dietary restraint was measured using the dietary restraint subscale of the EDE-Q (scores 0–6) [5] and the restrained eating subscale of the DEBQ (scores 1–5) [4]. The EDE-Q has four subscales—eating concern, weight concern, shape concern, and dietary restraint—and is validated for use in adolescents 14 years and over [5, 12]. Items in the dietary restraint subscale ask about attempts to limit dietary intake, following food rules, and the desire to have a flat stomach [5]. The DEBQ has three subscales—emotional eating, external eating and restrained eating—it is recommended for use in adolescents >12 years and has been validated in children as young as 9 years old [4, 13]. Items in the restrained eating subscale ask about attempts to limit dietary intake, food refusal, monitoring dietary intake, choosing ‘slimming’ foods, modifying dietary patterns, and the influence of weight on dietary choices.

Binge eating was assessed using the total score on the Binge Eating Scale (BES; scores 0–46) [14]. The BES is designed to assess binge eating severity and has been used in research with adolescents with obesity aged 12 years and older [14–16].

The changes in EDE-Q dietary restraint, DEBQ restrained eating, and BES total score between baseline and week-4, week-16, and week-52 were calculated.

#### Anthropometric outcomes and intervention adherence

Weight and height were measured by blinded trained assessors using standard procedures at baseline, week-4, week-16 and week-52 and used to calculate age- and sex-adjusted BMI z-score [10]. Changes in BMI z-score between baseline and week-4, week-16, and week-52 were calculated.

At 12 review appointments, dietitians recorded whether they perceived the participant to be ‘not following’ the meal plan, following it ‘sometimes’, ‘most of the time’ or ‘all of the time’. Participants were classed as ‘adherent’ to the meal plan if they were rated as following the plan ‘most’ or ‘all’ of the time during at least 75% of their attended dietetic reviews up to each timepoint (i.e. week-4, week-16, week-52).

### Statistical analysis

Analysis was conducted using IBM SPSS Statistics (V29). Participants with data available for any outcome of interest were included in the analysis. Dietary intervention groups were analysed together, as there was no difference between groups in anthropometric or psychosocial outcomes considered in this analysis [7–9]. Baseline characteristics of participants included and excluded from the analysis were compared using independent samples t-test or Mann–Whitney U test for continuous variables and Pearson’s Chi-squared tests for categorical data. Perceived adherence of participants included and excluded from analysis at each timepoint was compared using Pearson’s Chi-squared tests.

Pearson r correlation coefficient and Spearman’s rank correlation were used to examine the association between: EDE-Q and DEBQ restraint subscale scores at baseline, week-4, week-16, and week-52; change in dietary restraint (on the EDE-Q and DEBQ) and change in binge eating at week-4, week-16, and week-52; and change in dietary restraint and change in BMI z-score at week-4, week-16, and week-52. Mean or median change in dietary restraint on both tools was compared between perceived adherers and non-adherers at each timepoint using independent samples t-tests or Mann–Whitney U tests.

## Results

### Baseline characteristics

141 adolescents participated in the trial (median[interquartile range] age = 14.8[1.9] years, 49.6% female). Participants had a mean(standard deviation) BMI z-score of 2.40(0.46) and the most common cardiometabolic complications at baseline were dyslipidaemia (*n* = 60[42.6%]) and insulin resistance (*n* = 111[81.6%]) [7]. Most participants had at least one parent with obesity (*n* = 97[68.8%]) and/or a family history of diabetes (*n* = 117[83.0%]) [7] and 73% had tried at least one previous diet [17] (Supplementary Table 1). Of the 141 enrolled participants, 136 had data available to be included in baseline, 130 in week-4, 120 in week-16, and 92 in week-52 analysis. Participants included in the week-4 and week-16 analyses were significantly more likely to have been classified as adherent to the intervention based on dietitian report at these timepoints, than those excluded from analysis. A greater proportion of participants in the IER group were excluded from week-52 analysis than from the CER group. There were no other significant differences between baseline characteristics of participants included and excluded from analysis (Supplementary Table 2).

### Association between measures of dietary restraint

The EDE-Q dietary restraint and DEBQ restrained eating scores were positively correlated at each timepoint (week-0: *r* = 0.442, week-4: *r* = 0.503, week-16: *r* = 0.516, week-52: *r* = 0.579, all *p* < 0.001) (Supplementary Table 3).

### Association between dietary restraint and binge eating

There were no significant correlations between the change in EDE-Q dietary restraint from baseline to week-4, week-16 or week-52 and the change in binge eating (Table 1).

**Table 1:**
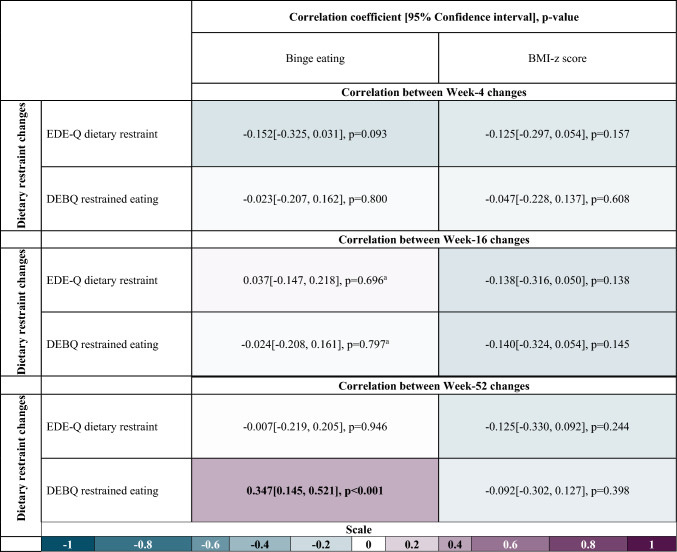
The correlation between the change in dietary restraint (as measured by the Eating Disorder Examination Questionnaire and Dutch Eating Behaviour Questionnaire) from baseline to Weeks-4, -16, and -52 and the change in binge eating and BMI z-score, Spearman’s rank correlation was used unless otherwise indicated in the footnote

BMI: body mass index; DEBQ: Dutch Eating Behaviour Questionnaire; EDE-Q: Eating Disorder Examination Questionnaire

^a^Pearson correlation used

There were no significant correlations between the change in DEBQ restrained eating from baseline to week-4 or week-16 and the change in binge eating. There was a positive correlation between the change in DEBQ restrained eating and change in binge eating from baseline to week-52 (*r* = 0.347, *p* < 0.001) (Table 1).

### Association between restraint, change in BMI z-score and perceived adherence

Changes in EDE-Q dietary restraint and DEBQ restrained eating from baseline to week-4, week-16 and week-52 were not significantly correlated with changes in BMI z-score at each timepoint (Table 1), nor were they significantly different between participants perceived as intervention adherers and non-adherers (Table 2).

**Table 2:** The difference between the change in EDE-Q and DEBQ dietary restraint between dietitian perceived adherers and non-adherers to the intervention at each timepoint

		Baseline to Week-4			Baseline to Week-16			Baseline to Week-52		
		Adherers (*n* = 107)	Non-adherers (*n* = 23)	Test statistic, *p*-value^d^	Adherers (*n* = 92)	Non-adherers (*n* = 28)	Test statistic, *p*-value^d^	Adherers (*n* = 55)	Non-adherers (*n* = 37)	Test statistic, *p*-value^d^
Dietary restraint changes	EDE-Q dietary restraint^a^ (mean change, SD)^b^	0.80(2.80)	1.00(2.40)	U = 1279.00, *p* = 0.767	0.67(1.51)	0.26(1.05)	t(118) = − 1.344, *p* = 0.181	0.44(1.56)	-0.01(1.63)	t(90) = − 1.327, *p* = 0.188
	DEBQ restrained eating^c^ (mean change, SD)	0.08(0.72)	0.12(0.85)	t(121) = 0.226, *p* = 0.822	0.32(0.83)	0.0009(0.81)	t(111) = − 1.729, *p* = 0.087	0.14(0.83)	0.15(0.70)	t(88) = 0.066, *p* = 0.948

DEBQ: Dutch Eating Behaviour Questionnaire; EDE-Q: Eating Disorder Examination Questionnaire; IQR: interquartile range; SD: standard deviation

^a^The dietary restraint subscale of the EDE-Q is scored on a scale of 0–6, with higher scores indicating higher levels of dietary restraint

^b^The change in EDE-Q dietary restraint is not normally distributed at Week-4, median(IQR) reported at this timepoint

^c^The restrained eating subscale of the DEBQ is scored on a scale of 1–5, with higher scores indicating higher levels of restrained eating

^d^The difference between perceived adherers and non-adherers was assessed using independent samples t-tests (for normally distributed variables) or Mann–Whitney U tests (for non-normally distributed variables)

## Discussion

In this study, we found that different measures of dietary restraint—the EDE-Q and DEBQ subscales—were positively correlated; however, the correlations were moderate to weak. We found no association between changes in dietary restraint and changes in binge eating at week-4, following the VLED, or at week-16, following the intensive phase of the dietary intervention. However, an increase in dietary restraint at week-52, measured using the DEBQ but not the EDE-Q, was associated with an increase in binge eating score. We found no association between the change in dietary restraint and change in BMI z-score or perceived adherence to the intervention.

The weak-to-moderate correlations between the measures of dietary restraint used in the study are somewhat surprising. Previous research in adults has examined whether different restraint measures capture different facets of dietary restraint, finding that both the DEBQ and EDE-Q dietary restraint items loaded onto the same facet—weight-focussed restraint—thus, we might expect the items to be more strongly correlated in the current analysis [18]. However, as discussed earlier, the EDE-Q and DEBQ were developed in different contexts. The DEBQ was specifically designed to characterise eating patterns associated with obesity and was developed in cohorts with and without overweight [4]. The EDE-Q was derived from and validated against the Eating Disorder Examination (EDE) interview [5]. This interview was initially developed and validated in community samples and clinical populations with anorexia nervosa or bulimia nervosa [5, 19]. The EDE subscales were developed based on rational grouping of items with similar meaning rather than empirically [20], and since their development, evidence regarding the validity of the factor structure of the EDE-Q is mixed [21]. It is possible that the items of these two subscales may be interpreted differently, given these contextual differences in their development.

Our findings suggest that increases in dietary restraint among adolescents engaged in an intensive weight management intervention are not strongly associated with changes in binge eating behaviours during the intervention. The lack of association between EDE-Q dietary restraint and binge eating is inconsistent with restraint theory, models of binge eating development, and evidence from community-based studies [1, 2]. It is noteworthy that dietary restraint scores peaked at week-4 on the EDE-Q and week-16 on the DEBQ [8, 9], yet at these timepoints an increase in dietary restraint did not appear to be associated with worsening binge eating behaviours. In contrast, at week-52, average dietary restraint scores on both scales had returned to baseline levels; however, there was a weak positive correlation between the change in DEBQ restrained eating and binge eating at this timepoint. These findings may suggest that acute increases in dietary restraint during a weight management intervention have a differential effect on disordered eating behaviours compared to sustained cognitive efforts to restrict intake. However, given the small sample size, relatively weak correlation, and number of correlations tested, it is possible this is a chance finding and thus should be interpreted with caution. This remains an important area for further research.

In this study changes in dietary restraint were not associated with weight change or perceived adherence to the intervention. Interestingly, the pattern of change in mean dietary restraint scores somewhat mirrored the pattern of weight change—with the greatest changes observed in the first 16 weeks of the intervention, which may be misinterpreted as the dietary restraint increases being related to weight loss. However, dietary restraint and weight change were not significantly correlated. These findings lend support to the argument that existing dietary restraint measures should not be interpreted as measures of actual caloric restriction, and that changes in dietary restraint may capture the *intent* to modify dietary intake to align with intervention recommendations [1]. Overall, the literature regarding the association between dietary restraint and weight outcomes remains mixed, with some studies suggesting that dietary restraint can support weight loss and weight loss maintenance [22] and others finding that individuals with higher dietary restraint have a less favourable response to behavioural weight management interventions [23, 24]. Future research should examine potential mechanisms underlying this heterogeneity.

Overall, our findings do not provide strong support for the role of dietary restraint either as a marker of adherence or as a risk factor for the development of binge eating in the context of an intensive behavioural weight management intervention. While the DEBQ restrained eating subscale was associated with binge eating at the end of the intervention, the relatively weak correlation identified does not provide robust evidence regarding the role of dietary restraint in the context of obesity treatment and suggests there may be opportunities to more effectively capture the cognitive processes that drive disordered restraint behaviours. 

### Strengths and limits

To date, the relationship between dietary restraint and binge eating has been most often explored in observational studies [25]. This was an exploratory secondary data analysis and adds to a limited body of literature exploring the relationship between dietary restraint and binge eating within the context of a prescribed dietary intervention. This is an important first step in understanding the implications of the increase in dietary restraint that is often observed during behavioural weight management interventions [3]. However, this study has several limitations. The small sample size limits the ability to draw conclusions from the results. At week-4 and week-16, a greater proportion of participants who were perceived to be adherent to the intervention were included in the analysis than those perceived to be non-adherent, this may have limited our ability to identify associations between changes in dietary restraint and perceived adherence to the intervention. In addition, there are challenges objectively assessing adherence to dietary interventions. As such, we relied on dietitians’ perception of participant adherence. This assessment was based on clinical judgement and may be susceptible to bias. At week-52, there was a greater proportion of participants from the IER group excluded from the analysis than the CER group. This was driven by a higher withdrawal rate in the IER group, largely due to not wanting to continue with the dietary pattern [7]. This may limit the generalisability of the findings to a variety of intensive dietary interventions. However, given that the findings do not provide strong support for existing theories, this work highlights a need to replicate such research with larger samples of treatment-seeking adolescents with obesity.

It would be of interest to consider longitudinal modelling to examine the temporal relationship between dietary restraint, disordered eating and adherence in the context of behavioural weight management interventions. Such models could also examine the influence of factors such as adverse childhood experiences, trauma, food insecurity, weight-related teasing, family history of obesity and EDs, history of dieting, and psychological support on the relationship between dietary restraint, disordered eating and adherence [26]. Larger studies may also consider measuring other disordered eating outcomes, particularly purging behaviours, which are theorised to be associated with dietary restraint. Such studies may also examine associations between specific items of dietary restraint subscales with adherence and disordered eating outcomes. In the current study, certain aspects of models of ED development that are theorised to precede dietary restraint and disordered eating were not directly measured, specifically body dissatisfaction and thin ideal internalisation, future research may consider examining the association between these outcomes, the development of dietary restraint, and disordered eating outcomes in young people seeking obesity treatment. In addition, future research should examine whether the relationship between dietary restraint, ED outcomes, and adherence to a dietary intervention differs when adolescents engage in self-directed dieting (rather than professionally supervised weight management interventions) to achieve weight loss.

## What is already known on this subject?

Dietary restraint is considered a risk factor for the development of eating disorders. However, in the context of behavioural weight management interventions, dietary restraint often increases or remains unchanged following interventions without worsening of other eating disorder outcomes. Thus, there is uncertainty regarding the role of dietary restraint in the context of behavioural weight management interventions.

## What this study adds?

The current study found that there were not strong associations between the change in dietary restraint during an intensive behavioural weight management intervention and changes in binge eating, BMI z-score and adherence to the intervention. The findings suggest further research is needed to understand the changes in dietary restraint that occur during weight management interventions. Future research may also explore alternate means of identifying maladaptive components of dietary restraint that confer greater risk of disordered eating outcomes.

## Supplementary Information

Below is the link to the electronic supplementary material.

Supplementary file1 (DOCX 41 KB)

## Data Availability

The datasets analysed during the current study are available from the corresponding author on reasonable request subject to additional ethics approval.
