# Supplementary material for: Dietary restraint, binge eating and adherence to an adolescent intensive behavioural weight management intervention: a Fast Track to Health sub-study
Source: Eat Weight Disord. 2026 Jun 4;31(1):73. doi: 10.1007/s40519-026-01876-y (PMC13451412; doi:10.1007/s40519-026-01876-y)
Supplement: Supplementary file 1 [file 40519_2026_1876_MOESM1_ESM.docx]

**Supplementary material**

**Article title:** “Dietary restraint, binge eating and adherence to an adolescent intensive behavioural weight management intervention: a Fast Track to Health sub-study”

**Journal:** Eating and Weight Disorders – Studies on Anorexia, Bulimia and Obesity

**Authors:** Eve T House, Natalie B Lister, Louise A Baur, Sarah P Garnett, Megan L Gow, Cathy Kwok, Hiba Jebeile, on behalf of the Fast Track to Health study team

**Corresponding author:** Eve T House, The University of Sydney, [eve.house@sydney.edu.au](mailto:eve.house@sydney.edu.au)

**Supplementary Table 1: Baseline clinical and demographic characteristics of participants in the Fast Track to Health randomised controlled trial (n=141)^a,b^**

|  | **All participants (n=141)** |
| --- | --- |
| Age, years, median(IQR) | 14.8(1.9) |
| Sex, n(%) female | 70(49.6) |
| Anthropometry, mean(SD) |  |
| Weight, kg | 100.4(16.5) |
| Height, cm | 2.6(0.5) |
| Waist circumference, cm | 107.5(11.6) |
| BMI, kg/m^2^ | 35.4(4.2) |
| BMI z-score | 2.4(0.5) |
| Cardiometabolic profile, n(%) |  |
| Systolic blood pressure >95^th^ percentile | 17(12.1) |
| Diastolic blood pressure >95^th^ percentile | 17(12.1) |
| Dyslipidaemia^c^ (n=137) | 60(42.6) |
| Elevated liver enzymes^d^ (n=139) | 37(27.0) |
| Insulin resistance^e^ (n=136) | 111(81.6) |
| Elevated fasting glucose^f^ (n=138) | 11(8.0) |
| Country of birth (n=134), n(%) |  |
| Australia | 117(82.9) |
| India | 6(4.3) |
| Vietnam | 2(1.4) |
| Other | 9(12.1) |
| Language spoken at home (n=138)^g^, n |  |
| English | 131 |
| Arabic | 24 |
| Vietnamese | 3 |
| Italian | 2 |
| Greek | 4 |
| Spanish | 2 |
| Hindi | 3 |
| Dari | 3 |
| Other | 16 |
| Parental marital status (n=138), n(%) |  |
| Single parent | 40(28.3) |
| Married/de facto | 98(69.5) |
| Household income (AUD) (n=134), n(%) |  |
| <$20,000 | 11(7.8) |
| $20,000-$40,000 | 8(5.7) |
| $40,000-$60,000 | 12(8.5) |
| $60,000-$80,000 | 24(17.0) |
| >$80,000 | 51(36.2) |
| Prefer not to say | 28(19.9) |
| Socioeconomic Indexes for Area (SEIFA, from postcode) (n=138) |  |
| Index of Relative Socio-Economic Disadvantage (IRSD) |  |
| Greater than 75% | 44(31.2) |
| 50-75% | 33(23.4) |
| 25-50% | 21(14.9) |
| Less than 25% | 40(28.4) |
| Index of Relative Socio-Economic Advantage and Disadvantage (IRSAD) |  |
| Greater than 75% | 50(35.5) |
| 50-75% | 32(22.7) |
| 25-50% | 25(17.7) |
| Less than 25% | 31(22.0) |
| Parent with obesity (n=118), n(%) | 97(68.8) |
| Family history of diabetes (n=138), n(%) | 117(83.0) |
| Number of diets trialled by adolescents prior to study enrolment (n=138), n(%) |  |
| 0 | 35(24.8) |
| 1 | 73(51.8) |
| 2 | 26(18.4) |
| 3 | 4(2.8) |

*Abbreviations:* AUD – Australian dollars, BMI – body mass index, IQR – interquartile range, SD – standard deviation

a. This table has been adapted from **Table 1** and **eTable 2** of Lister NB, Baur LA, House ET, Alexander S, Brown J, Collins CE, et al. (2024) Intermittent Energy Restriction for Adolescents With Obesity: The Fast Track to Health Randomized Clinical Trial. JAMA Pediatr 178(10):1006-1016. https://doi.org/10.1001/jamapediatrics.2024.2869 and **Table 1** of Jebeile H, House ET, Baur LA, Kwok C, Collins CE, Garnett SP, et al. (2025) Dieting Practices of Adolescents Seeking Obesity Treatment. Nutrients 17(19):3100. https://doi.org/10.3390/nu17193100
b. Percentages reported are based on the total number of participants (n=141), this may differ from the number of participants with data available for each variable
c. Defined as triglycerides>150mg/dL and/or HDL cholesterol <40mg/dL
d. Defined as alanine amino-transferase or γ-glutamyl transferase 1.5 times or more than the upper limit of 30U/L
e. Defined as fasting insulin:glucose ratio >20
f. Defined as 100-124mg/dL
g. Participants may speak more than one language at home

**Supplementary Table 2: Comparison of baseline characteristics and perceived adherence of participants included and excluded from the analysis at each timepoint**

|  | **Baseline analysis^a^** | | | **Week-4 analysis** | | | **Week-16 analysis** | | | **Week-52 analysis** | | |
| --- | --- | --- | --- | --- | --- | --- | --- | --- | --- | --- | --- | --- |
| **Characteristic** | **Included (n=136)** | **Not included (n=5)** | **Test statistic, p-value** | **Included (n=130)** | **Not included (n=11)** | **Test statistic, p-value** | **Included (n=120)** | **Not included (n=21)** | **Test statistic, p-value** | **Included (n=92)** | **Not included (n=49)** | **Test statistic, p-value** |
| **Baseline characteristics** | | | | | | | | | | | | |
| Age (median, IQR) | 14.75(1.90) | 15.66(2.25) | U=252.00, p=0.327 | 14.74(1.93) | 15.26(2.26) | U=648.00, p=0.607 | 14.85(1.76) | 14.36(2.23) | U=1481.50, p=0.200 | 15.05(1.86) | 14.58(1.85) | U=2548.00, p=0.203 |
| Sex (n, % female) | 68(50%) | 2(40%) | Χ^2^=0.193, p=1.000^b^ | 64(49%) | 6(55%) | Χ^2^=0.115, p=0.735 | 59(49%) | 11(52%) | Χ^2^=0.074, p=0.786 | 47(51%) | 23(47%) | Χ^2^=0.220, p=0.639 |
| Allocated dietary pattern (n, % IER) | 68(50%) | 3(60%) | Χ^2^=0.193, p=1.000^b^ | 65(50%) | 6(55%) | Χ^2^=0.084, p=0.772 | 59(49%) | 12(57%) | Χ^2^=0.455, p=0.50 | 40(43%) | 31(63%) | **Χ^2^=5.007, p=0.025** |
| BMI z-score (Mean, SD) | 2.38(0.46) | 2.79(0.46) | t(139)=1.959, p=0.052 | 2.38(0.45) | 2.56(0.53) | t(139)=1.264, p=0.208 | 2.39(0.45) | 2.45(0.55) | t(139)=0.577, p=0.565 | 2.38(0.44) | 2.43(0.50) | t(139)=0.578, p=0.564 |
| Dietary restraint (EDE-Q) (Median, IQR) | 1.00(1.80) | 1.40(1.33) | U=328.00, p=0.893 | 1.00(1.8) | 1.80(2.60) | U=650.00, p=0.616 | 1.00(1.84) | 1.40(1.90) | U=1235.50, p=0.887 | 1.00(1.75) | 1.20(2.05) | U=2104.50, p=0.516 |
| Restrained eating (DEBQ) (Mean, SD) | 2.50(0.81) | N/A | - | 2.51(0.80) | 2.38(1.03) | t(134)=-0.477, p=0.634 | 2.52(0.82) | 2.38(0.78) | t(134)=-0.719, p=0.474 | 2.48(0.81) | 2.54(0.82) | t(134)=0.378, p=0.706 |
| Binge eating (BES) (Median, IQR) | 11.00(12.00) | N/A^c^ | - | 11.00(11.50) | 11.29(15.75) | U=550.00, p=0.528 | 11.00(12.00) | 10.83(10.20) | U=1118.50, p=0.845 | 11.00(12.00) | 10.00(10.45) | U=1929.50, p=0.733 |
| **Adherence** | | | | | | | | | | | | |
| Classified as adherent at timepoint (n, % adherers) | N/A | N/A | - | 107(82%) | 6(55%) | **Χ^2^=4.912, p=0.042^b^** | 92(77%) | 11(52%) | **Χ^2^=5.354, p=0.021** | 55(60%) | 22(45%) | Χ^2^=2.858, p=0.091 |

*Abbreviations:* BES – Binge Eating Scale; BMI – body mass index; DEBQ – Dutch Eating Behaviour Questionnaire; EDE-Q – Eating Disorder Examination Questionnaire; IER – intermittent energy restriction; IQR – interquartile range; N/A – not applicable; SD – standard deviation

a. The analysis conducted with baseline data included the correlation between baseline scores on the EDE-Q dietary restraint and DEBQ restrained eating subscale, five participants did not complete the DEBQ at baseline
b. Fisher’s exact test used due to cells with expected cell count below n=5
c. Only one participant who was not included in the baseline analysis completed the BES, thus, the median score could not be calculated

**Supplementary Table 3: Correlation between different measures of dietary restraint: the Eating Disorder Examination Questionnaire (EDE-Q) and Dutch Eating Behaviour Questionnaire (DEBQ) restraint outcomes**

|  |  | **Correlation coefficient [95% Confidence interval], p-value** | | | |
| --- | --- | --- | --- | --- | --- |
|  |  | **DEBQ restrained eating** | | | |
|  |  | **Baseline** | **Week-4** | **Week-16** | **Week-52** |
| **EDE-Q dietary restraint** | **Baseline** | 0.442[0.291, 0.571], p<0.001^a^ |  |  |  |
|  | **Week-4** |  | 0.503[0.360, 0.623], p<0.001^b^ |  |  |
|  | **Week-16** |  |  | 0.516[0.369, 0.638], p<0.001^b^ |  |
|  | **Week-52** |  |  |  | 0.579[0.418, 0.704], p<0.001^a^ |

*Abbreviations:* DEBQ – Dutch Eating Behaviour Questionnaire; EDE-Q – Eating Disorder Examination Questionnaire

a. Spearman rank correlation
b. Pearson correlation
